# Comparative Evaluation of Breast Ultrasonography and Cytohistopathology in Diagnosing Breast Diseases

**DOI:** 10.7759/cureus.91478

**Published:** 2025-09-02

**Authors:** Tehzeeb Abdul Sattar, Abdullah Akram, Jahanzeb Akhtar, Sidra Shabbir, Narjes Sajjad, Abdullah Yousaf, Usman Mazhar, Mirza Zeeshan Sikandar

**Affiliations:** 1 Pathology, Liaquat University of Medical and Health Sciences, Jamshoro, PAK; 2 Oncology, Nawaz Sharif Medical College, Gujrat, PAK; 3 Pathology, Central Park Teaching Hospital, Lahore, PAK; 4 General Surgery, Rashid Latif Medical College, Lahore, PAK; 5 Oncology, Heavy Industries Taxila Education City-Institute of Medical Sciences (HITEC-IMS) Taxila Cantt, Taxila, PAK; 6 Medicine, Chughati Medical Centre, Gujranwala, PAK; 7 Primary and Secondary Health, Rural Health Center Mandi Ahmedabad, Mandi Ahmedabad, PAK

**Keywords:** benign breast lesions, breast cancer, breast ultrasonography, cytohistopathology, diagnostic accuracy

## Abstract

Introduction: Breast diseases include benign and malignant lesions, wherein benign ones are more common than malignant ones. Early differentiation is fundamental in low-resource environments.

Objective: The objective of this study is to determine the diagnostic accuracy of ultrasonography compared with cytohistopathology in differentiating benign and malignant breast lesions.

Methods: A cross-sectional study was conducted at the Pakistan Health & Research Forum between July 2024 and January 2025. One hundred and twenty-five newly diagnosed cases of breast lesions that had been treated with both ultrasonography and cytohistopathology assessment were included. Parameters of diagnostic accuracy, such as sensitivity and specificity, predictive values, and accuracy, were determined. To test the view of agreement, the chi-square and the McNemar tests were employed.

Results: Out of 125 participants (mean age: 43.8 with a standard deviation of 12.4 years), the histopathology of the tumor of 71 participants (56.8%) showed malignant lesions, and 54 participants (43.2%) had benign lesions. The sensitivity was 92.2 percent, specificity 96.7 percent, positive predictive value 96.7 percent, negative predictive value 92.2 percent, and diagnostic accuracy 94.4 percent. The results revealed a statistically significant correlation between malignancy and the increase in age (p < 0.001). There was a significant association in the chi-square test (p < 0.001) and in the McNemar test showing the diagnostic agreement (p = 0.453).

Conclusion: Ultrasonography is highly reliable in terms of diagnostics and can be used as a first-line procedure when making a distinction between benign and malignant lesions in the breast, especially where histopathology is not readily available. It can also become a part of the clinical practice to improve early diagnoses and management.

## Introduction

Breast diseases can be classified into benign and malignant based on clinical, histological, and imaging modalities. Benign breast diseases are more common than their malignant counterparts [1-3]. Benign diseases are sub-classified into inflammatory and epithelial lesions. Inflammatory lesions further comprise mastitis and duct ectasia, whereas epithelial lesions comprise proliferative and nonproliferative lesions and atypical hyperplasia [4,5]. Proliferative lesions include sclerosing adenosis and papilloma, whereas nonproliferative lesions include cystic changes with apocrine metaplasia, fibrosis, and adenosis [6,7]. Keeping in view the malignant counterparts, they are broadly divided into ductal and lobular carcinoma, with ductal carcinoma being the most common of the two.

According to the latest research, the prevalence of breast cancer in Pakistan is estimated to be 31.5% and rising [4]. Breast cancer carries a high mortality rate among the female population; therefore, an accurate diagnostic modality is crucial for timely and effective management [7,8]. Although ultrasound is not considered the gold standard in terms of diagnostics, it is widely used in initial investigations because of its easy accessibility and safety [9,10]. It is particularly useful to examine dense breast tissues, which appear obscured on the mammogram [11,12]. The absence of ionizing radiation makes ultrasound a relatively safer diagnostic modality in pregnant and lactating women. It is preferred among patients at high risk of breast carcinoma for whom an MRI is contraindicated [13].

Early identification and characterization of breast lesions are important for guiding clinical management, especially in resource-poor environments [7]. In this respect, the imaging process can be seen as instrumental in enabling the distinction between suspicious lesions, which necessitate further biopsy treatment, and those that can be stably observed. Although mammography is widely used as a screening method, it has poor sensitivity, especially in thick breast tissue among women who are young and thin [6,8]. Ultrasound is used more as a supplement to mammography, and it is characterized by imaging with high resolution, visualization in real time, and a better work rate in some clinical settings. Additionally, it is noninvasive and painless and does not expose a person to radiation [9]. Conversely, cytohistopathological examination involves tissue sampling and therefore is invasive and not always widely accessible in an ordinary clinical specification. Hence, assessment of the diagnostic ability of breast ultrasound against cytohistopathology is indispensable in clarifying its significance both as an independent and a supplementary diagnostic method in a situation where pathology services are scarce or delayed. The current study will determine the diagnostic accuracy of ultrasonography compared with cytohistopathology in differentiating benign and malignant breast lesions. The results will help assess the practical utility of ultrasound and cytohistopathology in diagnosing breast diseases.

## Materials and methods

After obtaining ethical approval (PHRF/2024/2404), a comparative cross-sectional study was conducted on the utility of ultrasonography and cytohistopathology in the diagnosis of breast disease at Pakistan Health & Research Forum from July 2024 to January 2025. A sample size of 125 was calculated while maintaining 80% power of test and 5% level of significance. A 21.4% prevalence rate of breast cancer in women was expected [12]. Only newly diagnosed breast disease patients who had been diagnosed over the period of the last six months were included by employing the random sampling technique, whereas those with previous metastasis or other diseases and secondary causes of breast cancer like endometrial carcinoma and lymphomas were excluded, and men having breast carcinoma were also excluded.

Ultrasounds of patients conducted on Aplio 300 (Canon Medical Systems Corporation, Tochigi, Japan) using a linear probe of 7.5 MHz were collected from the hospital’s radiology department. Likewise, the cytohistopathology samples, which were formalin-fixed tissue blocks stained with hematoxylin and eosin, were collected from patients in the pathology department with the corresponding MR number on the ultrasound report. The sensitivity and specificity of the ultrasound were assessed, keeping cytohistopathology as the gold standard.

Statistical analysis

IBM SPSS Statistics for Windows, Version 26 (Released 2019; IBM Corp., Armonk, New York, United States) was used to analyze data. Continuous data were represented by mean and standard deviation, and frequencies and percentages were applied to categorical data. Assessment of normality was performed using the Shapiro-Wilk test. An independent sample t-test was conducted for testing means between benign and malignant groups. Sensitivity, specificity, positive predictive value (PPV), negative predictive value (NPV), and accuracy of the ultrasonography were calculated against the gold standard: cytohistopathology. The chi-square and McNemar tests were used for the assessment of the relationships between ultrasound and histopathology results. At < 0.05, p-values were considered to be statistically significant.

## Results

A total of 125 patients participated in the study. The mean age was 43.8 + 12.4 years, with a minimum age of 18 and a maximum of 75. Most patients who had benign lesions were younger, with a mean age of 35.8 + 11.6 years, and most patients who had malignant lesions were older, with a mean age of 50.0 + 9.2 years (Table 1).

**Table 1:** Independent Sample T-Test Results of Comparison of Mean Age Between Participants with Benign and Malignant Lesions

Group	Mean Age (Years)	SD	n	T-statistic	P-value	Significance
Benign Lesions	35.8	11.6	54	7.47	< 0.001	Significant
Malignant Lesions	50.0	9.2	71			

Most of the participants (45, 36.0%) were between 40 and 49 years, and 34 (27.2%) were between 30 and 39 years. The majority of patients lived in cities (79, 63.2%) and had at least an intermediate-level education (65, 52%). In terms of socioeconomic status, more than half (63, 50.4%) were in the middle-income bracket, 32 (25.6%) were in the low-income bracket, and 30 (24%) were in the high-income bracket. A total of 29 (23.2%) respondents had a family history of breast cancer. The majority of women were married (113, 90.4%) and parous (105, 84.0%), whereas 20 (16.0%) were nulliparous (Table 2).

**Table 2:** Sociodemographic Characteristics of Study Participants (n = 125)

Variable	Categories	Frequency (n)	Percentage (%)
Age (years)	< 30	18	14.4
	30–39	34	27.2
	40–49	45	36.0
	≥ 50	28	22.4
Residence	Urban	79	63.2
	Rural	46	36.8
Educational Status	Illiterate	21	16.8
	Primary to Matric	39	31.2
	Intermediate or Above	65	52.0
Socioeconomic Status	Low	32	25.6
	Middle	63	50.4
	High	30	24.0
Family History of Breast Cancer	Present	29	23.2
	Absent	96	76.8
Marital Status	Married	113	90.4
	Unmarried/Widowed	12	9.6
Parity	Nulliparous	20	16.0
	Parous	105	84.0

Among the 125 patients who received a histopathological diagnosis, 71 (56.8%) had malignant lesions, and 54 (43.2%) had benign lesions. Invasive ductal carcinoma was reported as the malignant diagnosis in 61 patients (85.9%), and ductal carcinoma in situ and other malignancies were reported in seven patients each (7.0%). The most reported lesion in the benign group was fibroadenoma (23, 42.6%), followed by breast cysts (8, 14.8%), breast abscesses (6, 11.1%), and other benign lesions (17, 31.5%) (Table 3).

**Table 3:** Distribution of Histopathological Lesion Types (n = 125)

Lesion Type	Frequency (n)	Percentage (%)
Benign Lesions		
Fibroadenoma	23	42.6%
Breast Cyst	8	14.8%
Breast Abscess	6	11.1%
Other Benign Lesions	17	31.5%
Total Benign Lesions	54	100.0%
Malignant Lesions		
Invasive Ductal Carcinoma	61	85.9%
Ductal Carcinoma in Situ (DCIS)	5	7.0%
Other Malignant Lesions	5	7.0%
Total Malignant Lesions	71	100.0%

Sixty-one patients had malignant signs according to the ultrasonographic assessment, and 64 had benign signs. The cross-referencing between the ultrasound and cytohistopathology indicated that in the diagnosis of the malignant and nonmalignant lesions, the true positive diagnosis was that of 59 malignant lesions, and the true negative diagnosis was that of 59 benign lesions. The percentage of false positives was 2% and that of false negatives was 5%, which corresponded to an overall diagnostic accuracy of 94.4%. The sensitivity of ultrasonography was 92.2%, the specificity was 96.7%, the PPV was 96.7%, and the NPV was 92.2% (Table 4).

**Table 4:** Diagnostic Accuracy Indices of Ultrasonography (n = 125)

Index	Value (%)
Sensitivity	92.2%
Specificity	96.7%
Positive Predictive Value	96.7%
Negative Predictive Value	92.2%
Accuracy	94.4%

The chi-square test was statistically significant (p < 0.001), demonstrating consistency between the ultrasonography and cytohistopathology results. The McNemar test demonstrated no significant disagreement between paired diagnostic classifications (p = 0.453), indicating that two modalities were of high consistency (Table 5).

**Table 5:** Diagnostic Performance of Breast Ultrasound Compared to Cytohistopathology (n = 125)

Ultrasound Result (n = 125)	Histopathology Malignant	Histopathology Benign	Chi-Square (χ²)	P-Value	McNemar’s Test P-Value
Malignant (Positive) (n = 61)	59 (True Positive)	2 (False Positive)	106.74	< 0.001	0.453
Benign (Negative) (n = 64)	5 (False Negative)	59 (True Negative)			

Age-stratified analysis indicated that there was a significant association between an increase in age and the possibility of developing malignant lesions. In patients aged more than 50 years, malignant findings were present in 36 (90%) patients. In comparison, 10 malignancies were present in 14.1% of patients aged between 18 and 35 years. This correlation was statistically significant (width = 39.82, p < 0.001), indicating a strong dependence of the malignancy of breast lesions on age (Table 6).

**Table 6:** Association of Age Groups With Malignancy Status

Age Group (Years)	Malignant (n = 71)	Benign (n = 54)	Total (n = 125)	Chi-Square	P-Value	Significance
18–35	10 (14.1%)	38 (70.4%)	48	χ² = 39.82	< 0.001	Significant
36–50	25 (35.2%)	12 (22.2%)	37			
> 50	36 (50.7%)	4 (7.4%)	40			

## Discussion

Breast ultrasonography has become a valuable method for diagnosing breast lesions because it is noninvasive, cheap, and does not expose patients to harmful ionizing radiation. In this research, ultrasonography showed a sensitivity of 92.2%, a specificity of 96.7%, a PPV of 96.7%, an NPV of 92.2%, and a total diagnostic precision of 94.4%. Such results support its usefulness, especially to distinguish between benign and malignant lesions in the case of a resource-poor environment.

Diagnostic accuracy has been discussed in the past, with diverse degrees of accuracy reported. In the meta-analysis by Zhang et al., the pooled sensitivity and specificity were 89% and 84%, respectively. These figures aligned to a large degree with the current analysis results [13]. Analogously, Tadesse et al. focused on the fact that operator experience has a great impact on diagnostic precision, particularly in terms of differentiating between complicated and benign lesions and malignancies [14]. The difference in the level of specificity experienced in the current study can be credited to the overlapping sonographic signs of some of the benign conditions, such as fibroadenoma and duct ectasia with malignant lesions, a phenomenon that was described by Ramala et al. [15].

The sensitivity in the current study was high, indicating that ultrasonography is useful in screening and excluding malignancy. This was in line with Uematsu et al., who reported that real-time B-mode ultrasound can successfully identify malignant masses at a sensitivity level of 90% or more [16]. The reduced specificity, however, suggests that false positive results are still a possibility and can result in unwarranted or uneasiness in the patient. The same restriction has been found in other contrastive trials like those by Nowikiewicz and Afzal et al. These authors recommended ultrasound usage along with mammography or elastography to achieve an enhanced level of diagnostic accuracy [17,18].

The application of fine needle aspiration (FNAC) and cytohistopathology as confirmatory practices allowed stable contrast. The final description was fulfilled by histopathology as the gold standard and FNAC as a rapid cytological reproduction. Triple assessments involving clinical examination, imaging, and cytopathology are important for diagnosing breast diseases and remain the mainstay in this field [19].

Although the current study, by using sampling covering various kinds of lesions and demographic groups, is based on a strong foundation, it has certain limitations. It is possible that its single-center design reduces its generalizability. Further, blinded interpretations of ultrasound were not conducted, which might have introduced bias. Moreover, the generalizability of ultrasonic findings beyond being operator-dependent must be acknowledged. A multicenter study should be conducted in the future while ensuring the blinding process for generalizability of findings with a larger sample size and by adding a comparison to the male gender. Nevertheless, the current study’s results can be added to the existing evidence base supporting the application of ultrasound in primary diagnostic settings, particularly settings where mammography or MRI is not available at high frequency [20,21].

## Conclusions

Ultrasonography showed good sensitivity and fair specificity in the diagnosis of breast lesions, thus indicating its efficiency and convenience as a primary diagnostic method. Although cytohistopathology is still the gold standard for obtaining a definite diagnosis, some of the main benefits of ultrasound are that it is noninvasive, readily accessible, and economical. Additionally, it does not involve ionizing radiation, which is critical in resource-scarce settings and for vulnerable groups like pregnant/lactating women. Ultrasonography used in conjunction with clinical examinations and cytological assessments as a triple diagnostic modality majorly contributes to increasing patients’ confidence in the lesions’ diagnosis and management. Although its performance level is high, the problems of operator dependency and the similar appearance of malignant and benign lesions must be considered. A standardized imaging criterion and training should be offered to ensure a higher level of diagnostic accuracy. A multicentric study should be conducted in the future with more patients and broader populations to narrow down ultrasonographic criteria and formulate clear recommendations for whether and when ultrasonography can be used as an adjunct or alternative function in breast cancer diagnostic algorithms.
